# Supplementary material for: Dual IRE1 RNase functions dictate glioblastoma development
Source: EMBO Mol Med. 2018 Jan 8;10(3):e7929. doi: 10.15252/emmm.201707929 (PMC5840541; doi:10.15252/emmm.201707929)
Supplement: Supplementary file 1 — Appendix [file EMMM-10-e7929-s001.pdf]

APPENDIX - Supplementary Files

Table of Content

**Supplementary Materials and Methods**	----------------------------------	**Page 2**
**Supplementary Tables**	----------------------------------	**Page 7**
Table S1	-	Page 7
Table S2		Page 8
Table S3		Page 9
Table S4		Page 31
Table S5		Page 32
Table S6		Page 33
Table S7		Page 34
Table S8		Page 36
**Supplementary Figure Legends**	----------------------------------	**Page 37**
**Supplementary References**	----------------------------------	**Page 40**
**Supplementary Figures**	----------------------------------	**Page 42**
Figure S1		Page 42
Figure S2		Page 43
Figure S3		Page 44
Figure S4		Page 45
Figure S5		Page 46
Figure S6		Page 47
Figure S7		Page 48
Figure S8		Page 49

A) Supplementary Materials and Methods

Antibodies – All the antibodies used in this study are described in **Appendix Table S8**.

Biomass analysis (SRB) - The SRB assay was performed as previously described (Vichai & Kirtikara, 2006). Briefly, cells were seeded into 96-well plates in 100 µL at a density of 5000 cells/well. After cell inoculation, the plates were incubated at 37°C with 5% CO2 for 24, to 96 hrs. Cell were then fixed *in situ* with trichloroacetic acid and stained with sulforhodamine B (Sigma). Absorbance was measured at 510 nm.

Sphere formation and migration - Sphere formation experiments were performed by incubating 5000 cells by well in a 96-wells plate previously coated with 50 µl of 1.5% agar gel. For sphere dissociation/migration, spheres of the same size (obtained by incubating 5000 of each cell types for 48 hrs) were put on a 22-mm coverslip (Rempel, 2001) and incubated for 48 hrs. Then, spheres were fixed with 4% paraformaldehyde for 20 min at room temperature. Cell actin (phalloidin-FITC) and nucleus (Hoechst) were thus stained and visualized as previously described (Dejeans et al, 2012). Two parameters were measured: the size of the resulting sphere after 48 hrs of cell migration from the sphere, and the migrating distance of cells from the center of each sphere.

In vitro IRE1-mediated RNA cleavage and systematic analysis of the cleavage sites - Total RNA was extracted from U87 cells using Trizol reagent (Invitrogen, Carlsbad, CA, USA) and processed as: **1)** RNA was cleaved *in vitro* by recombinant IRE1 protein. The cleavage was repeated twice, using two different recombinant IRE1 (11905-H2OB, Sino Biological and E31-11G, SignalChem). The cleavage was performed for 4h at 37°C in 250 mM Tris pH 7.5, 100 mM NaCl, 5 mM MgCl2, 10 mM ATP, 0.33 µg/µl mRNA and 1 µg IRE1 protein per µg RNA. Alternatively, **2)** RNA was treated as for 1) in the absence of IRE1. PolyA and non-polyA RNA were then separated from the cleaved or uncleaved conditions using Dynabeads® mRNA DIRECT™ Purification Kit (Ambion) following provider's instructions. All steps were validated using RT-qPCR and RT-PCR against 3' or 5' specific primers of IRE1 mRNA targets (PER1 and XBP1) or against mRNA (GAPDH, B2M) and non-messenger RNA (18S ribosomal RNA, 7SK RNA, U1 spliceosomal RNA) (not shown). Biotinylated single strand cDNA was then prepared according to the WT AMBION kit manual RevD (4425209 RevD) from 250 ng total RNA and WTGene Affymetrix manualRev7 (P/N 702808 Rev7). Following fragmentation and terminal labeling, 5.5 ug of single strand cDNA were hybridized for 16h at 45°C on Affymetrix GeneChips. GeneChips were washed and stained in the Affymetrix Fluidics Station 450 with HWS kit and scanned using the Affymetrix

GeneChip Scanner 3000 7G. Data were generated with Affymetrix Expression Console v 1.2.1. The microarray platform utilized for the identification of potential targets of IRE1 *in vitro,* was the Affymetrix GeneChip Human Transcriptome Array (HTA) 2.0, which covers the whole exonic regions of the human genome, as well as junction regions between two adjacent exons. Four conditions were examined in total. The first one (A) included all Non-PolyA transcripts plus Non-PolyA fragments produced by PolyA transcripts that might be cleaved by IRE1. The second (B) included all PolyA transcripts plus PolyA fragments produced by potential cleavage from IRE1. The third condition (C) included all Non-PolyA transcripts and finally the fourth (D) referred to all PolyA transcripts. Two replicate samples for each condition were hybridized on Affymetrix array. All probes were arranged into probe sets that translated and summarized the data into gene-level, exon-level and splice–junction probe sets. The respective *.CEL files, were processed by Affymetrix Expression Console Software (version 1.4; Build 1.4.1.46) (Affymetrix (2015) Affymetrix Expression Console Software (version 1.4; Build 1.4.1.46; http://www.affymetrix.com/estore/browse/level_seven_software_products_only.jsp?productId=131414#1_1), selecting the Exon Level Analysis procedure. Raw probe intensities were summarized into PSR (Probe Selection Region) and Junction expression indices, using a standard procedure of GC background correction (Affymetrix (2005) Exon array background correction. http://media.affymetrix.com/support/technical/whitepapers/exon_background_correction_whitepaper.pdf), median-scaling normalization (4), and median-polish summarization (GC-RMA) (5). Then, some kind of alternative splicing analysis for PSRs and junctions between the different conditions was conducted using the Affymetrix Trancriptome Analysis Console (TAC) (v3.0) (http://www.affymetrix.com/estore/browse/level_seven_software_products_only.jsp?productId=prod760001#1_1) which permits visualization of data, depiction of expression changes at the gene and exon level as well as drill down into alternatively spliced exons. The presence of PSRs and junctions per sample was tested by DABG (Detection Above Background algorithm). An PSR/junction was expressed in a condition when >=50% samples had DABG<0.05. Intensity rates for any PSR or junction in a condition were computed using Tukey's Bi-weight Average Algorithm (Affymetrix (2002) Tukey's Bi-weight Average Algorithm (http://media.affymetrix.com/support/technical/whitepapers/sadd_whitepaper.pdf) and were compared statistically between two experimental conditions using One-way Between-Subject ANOVA. Fold change cut-off was adopted at the level of 1.5-fold in linear-natural scale, with a parallel ANOVA p-value threshold of 0.05. Two complementary types of comparisons were performed and their overlapping findings were identified. Transcripts, whose PSRs and junctions were characterized as present or upregulated from a specific

PSR to their end, based on the 1st comparison (A versus C condition) but they were absent or downregulated based on the 2nd comparison (B versus D condition), respectively, were considered as potential targets of IRE1.

Transcriptome analyses – For cellular analyses, total RNA was extracted using the NucleoSpin RNA II, total RNA isolation kit (Macherey-Nagel, Düren, Germany), from U87EV, U87DN, U87WT, U87S769F, U87Q780* cells. Samples were then analysed using Affymetrix arrays (kit HG U133+) at the Plateform "Puces à ADN THD" in Montpellier. The array intensity signals were analyzed fusing the libraries of Gene ARMADA software. Pre-processing was performed with the RMA method and the normalization was performed with the Quantile method. From the normalized values the Coefficient of Variation (CV) was estimated for each probe. In order to derive differentially expressed probes, the cells transfected with the empty vector represented the control category and statistical selection was based on the scaled CV method with the following criteria: fold change >|1| and CV_{scaled} > 2. In order to perform functional pathway analysis on the sets of differentially expressed genes, derived from the aforementioned statistical comparisons, we exploited the *BioInfoMiner* platform (Chatziioannou, 2011; Moutselos et al, 2010; Moutselos et al, 2011; Pilalis & Chatziioannou, 2013), which performs statistical and network analysis on various biological hierarchical vocabularies (Gene Ontology (GO) (Ashburner et al, 2000), Reactome pathways (Croft et al, 2013), the Human Phenotype Ontology (HPO) (Kohler et al, 2013) and MGI Mammalian Ontology (Eppig et al, 2015)) aiming to detect and rank significantly altered biological processes and the respective driver genes linking these processes. The *Bioinfominer* pipeline is available online at the website: https://bioinfominer.com. Hierarchical clustering was performed on the union of the differentially expressed probes from all the samples using complete linkage method and cosine distance metric.

For tumor analyses, a local cohort of 119 GBM patients treated with radiotherapy and concurrent/adjuvant temozolomide in accordance with the standard of care (GBMmark) was retrospectively recruited and used for transcriptome analysis. GBM specimens were obtained after informed consent from patients admitted to the neurosurgery department at Rennes University Hospital for surgical resection in accordance with the local ethic committee. Tumors used in this study were histologically diagnosed as grade IV astrocytoma according to the WHO criteria. Tumor samples were snap-frozen immediately after resection. All samples presented at least 70% of tumor cells. The extent of surgery was evaluated with an enhanced magnetic resonance imaging (MRI) performed within 24 hours after the resection. Total RNA was isolated with the NucleoSpin RNAII Kit (Macherey-Nagel, Hoerdt, France). RNA integrity (RNA Integrity Number ≥ 8) was confirmed with an Agilent 2100 bioanalyzer (Agilent Technologies, Les Ulis, France). Gene expression profiling was

carried out with the Agilent whole human genome 8x60K microarray kit (Agilent Technologies). Total RNA was extracted, labelled and hybridized according to the kit manufacturer's recommendations. Raw intensity data were log2-transformed and normalized (intra-array and inter-array scaling) using *GeneSpring* software (Agilent Technologies). Student *t*-tests with a Welch approximation were used to compare expression values between conditions. Adjusted *p* values were calculated by controlling for the false discovery rate with the Benjamin i& Hochberg procedure. Genes were considered significantly differentially expressed if the *p* value was below 0.05. mRNA expression data were assessed from the publicly available GBM dataset of The Cancer Genome Atlas (TCGA) (Consortium et al, 2007; consortium, 2008) from the NCBI website platform https://gdc-portal.nci.nih.gov/.

Patients were clustered according to IRE1 activity based on the normalized z-score of gene expression for the *BioInfoMiner* exported, signature of 38 genes (Figure S1). The z-score was calculated by the equation (*X* - m)/s, *X* stands for normalized log2 expression data of each gene in each sample; m stands for mean of expression of each gene among all samples; and s stands for its respective, standard deviation. Raw data (*.CEL files) of GSE27306 dataset (https://www.ncbi.nlm.nih.gov/geo/query/acc.cgi?acc=GSE2730) from Pluquet et al.,2013 were processed into R/Bioconductor according to the RMA normalization and Limma package (Ritchie et al, 2015). The differentially expressed genes (DEGs) between DN and WT U87 cells, were selected by setting a corrected P value threshold of 0.05 and fold change one of |log2(fc)|≥1.5. In this way, 1051 differentially expressed (D.E.) genes were derived, which were then introduced into *BioInfoMiner* and gene prioritization was executed exploiting the ontological vocabularies of four, different, functional and phenotypic databases; GO, Reactome, MGI and HPO, separately. Moreover, their annotations were corrected for potential, semantic inconsistencies of their schema, by adopting the "complete" choice (this version links the annotation of each gene with the ancestors of every direct correlated ontological term, thus restoring the flawless structure of ontological tree) whereas a hypergeometric p-value threshold of 0.05, was adopted. This yielded 227 highly prioritized genes, including their proximal interactors as the result of the union of the BioInfoMiner output from the four databases, 38 of which were cherry-picked as the intersection with the IRE1 signature of 97 genes identified in (Pluquet et al, 2013) (GSE27306). Overall, the *BioInfoMiner* signature comprised 19 highly up-regulated genes in WT versus DN U87 cells (*ASS1, C3, CCL20, COL4A6, CXCL2, CXCL5, CXCL8, IFI44L, IL1B, IL6, KCNN2, MMP1, MMP12, MMP3, PLA2G4A, PPP4R4, SERPINB2, TFPI2, ZNF804A*), and 19 highly down-regulated genes in WT versus DN U87 cells (*ANGPT1, CFH, CFI, CLEC3B, COL3A1, COL8A1, DACH1, DCN, FHL1, GAS1, LUM, OXTR, PLAC8, RGS4, TAGLN, TGFB2, THBS1, TIMP3, TMEM255A*). These two components of

BioInfoMiner signature were also used for the expansion of XBP1s and RIDD signature of 40 genes (**Fig 4B**) and 37 genes (**Fig 4G**), respectively, for the evaluation of XBP1s and RIDD activity in patients.

For each patient, each gene of the “XBP1s” and “RIDD” signature respectively, was initially assigned a quartile-oriented, gene score according to the level of its expression when contrasted to its complete expression distribution in the specific cohort. Each gene of the signature was rated with 1 when the z-score was < = Q1(the first quartile; the 25th item of ordered data); with 2 when the z-score was >Q1 AND < = Q2 (median); with 3 when the z-score was >Q2 AND < Q3 (the third quartile; the 75th item of ordered data) and with 4 when the z-score was > = Q3. After quartile ranking, each patient was assigned an “XBP1s” and “RIDD” score based on the average of gene scores for XBP1s genes and RIDD genes, respectively. The median of “XBP1s” and “RIDD” patient score divided the specific cohort into four groups; XBP+, XBP1s- and RIDD+, RIDD- groups. Patients with XBP1s and RIDD score >= Q3 were collected as XBP1s high and RIDD low patients, respectively, and patients with XBP1s and RIDD score < = Q1 were collected as XBP1s low and RIDD high patients, respectively, for survival analysis. Patients were clustered according to IRE1, XBP1s and RIDD activities using hierarchical clustering, generated in R environment (R version 3.4.1 for windows) using the ComplexHeatmap R package (https://github.com/jokergoo/ComplexHeatmap). The euclidean, complete distance method was used for the grouping of GBM patients (TCGA and GBMmark) into four categories; XBP1s+/RIDD+, XBP1s+/RIDD-, XBP1s-/RIDD+ and XBP1s-/RIDD-, based on their patient score. Messenger RNA expression levels of immune (IBA1, CD14, CD45 and CD164), angiogenic (CD31, vWF) and invasive (RHOA, CYR61 and CTGF) markers were compared between high and low groups. mRNA expression was considered significantly differentially expressed if the *p*-value was below 0.05 using GraphPad Prism software.

B) *Supplementary tables*

	REACTOME TERM	Number of DE Genes S769F	Q780*	P336L	A414T	WT	DN	Genes/ Term
Extracellular matrix organization	Extracellular matrix organization	8*	-	**16****	**33****	**13****	**48****	267
	Degradation of the extracellular matrix	-	-	**11****	**13****	6*	**23****	118
	Syndecan Interactions	3*	-	4*	-	-	-	27
	Collagen formation	-	-	**9****	-	-	15*	85
	Collagen degradation	-	-	7*	-	-	12*	63
	Non-integrin membrane-ECM interactions			6*	-	-	-	59
	Integrin cell surface interactions	-	-	7*	**12****	-	18*	83
	Elastic fibre formation	-	-	-	7*	-	-	44
	Laminin interactions	-	-	4*	5*	-	-	30
Signal Transduction	Signaling by retinoic acid	-	-	-	-	-	9*	
	Regulation of KIT signaling	-	-	-	3*	-	-	12
	Gastrin-CREB signalling pathway via PKC and MAPK	-	-	-	-	15*	-	409
	G alpha (q) signalling events	-	-	-	-	8*	-	187
Homeostasis	Homeostasis	-	-	-	31*	-	63*	521
	Common pathway of fibrin clot formation	-	-	-	-	4*	-	22
	Cell surface interactions at the vascular wall	-	-	7*	9*	-	15*	98
	Basigin interactions	-	-	4*	-	-	-	25
Immune System	Interleukin-1 processing	-	2*	3*	3*	**3****	-	7
	Interferon signaling	-	-	-	-	-	27*	180
	Endosomal/Vacuolar pathway	-	-	-	-	-	**7****	11
	Antigen Presentation:Folding, assembly and peptide loading of class I MHC	-	-	-	-	-	7*	25
	Trafficking and processing of endosomal TLR	-	-	-	-	-	5*	13
	Nucleotide-binding domain, leucine rich repeat containing receptor signaling pathways	-	-	-	-	-	10*	51
[illegible] m of lipids and lipoproteins	Bile acid and salt metabolism				5*	-		34
	Synthesis of the bile acids and bile salts	3*	3*	3*			7*	26
	Synthesis of Prostagladins and Thromboxanes	2*	2*	-	-	-	-	14
[illegible] olism of vitamins,	Retinoid metabolism and Transport	3*	3*	-	-	-	-	42
	Vitamin B1 metabolism	-	-	-	2*	-	-	4
Metabolism of proteins	Regulation of Insulin-like GF transport and uptake	2*	2*	-	4*	-	-	21
	Post-translational protein modification	-	-	-	23*	-	-	398
	Asparagine N-linked glycosylation	-	-	-	14*	-	-	181

Table S1: Functional enrichment in signaling pathway as deduced from the transcriptome analysis. * indicates p < 0.05; ** p < 0.01. Values reflecting an even more significant enrichment were indicated in bold.

Table S2: XBP1s regulated genes based on transcriptome analyses

**XBP1s sign**
ANAPC10
ARG2
BAMBI
CADPS2
CCDC109B
CD70
CISD2
COL5A2
CSTA
DKFZP564O0823
DNAJB9
DTNBP1
EZR
FAT
FGF2
FKBP11
GALC
GLUL
HMGN3
IL20RB
LAYN
LOC284561
MAPKAP1
NAP1L5
NME5
PAPSS2
PGM3
PPAPDC1B
PPP1R14C
PTER
RNASE4
SEC23B
SEC24D
SEC31A
SERP1
SLFN11
SPCS3
SRGN
STOX2
TMEM200A

Table S3: List of mRNA cleaved by IRE1 *in vitro*

A-B	C	D-F	G-J	K-L	M-N	O-Q	R	S	T	U-Z
AAK1	C12orf73	DAGLB	G6PD	KCNAB2	MAB21L1	OBSCN	RAB3D	SBF2	TAB1	UBE2D3
ABCA1	C15orf41	DBN1	GALK1	KCNH3	MACF1	OCM2	RAB3GAP2	SBF2-AS1	TACC1	UBE2F
ABCA3	C16orf89	DCBLD2	GALNT3	KCNH4	MAD1L1	OLFML2B	RAB5A	SCARB2	TAF2	UBE2G1
ABCB4	C17orf53	DCLK1	GANAB	KCNJ5	MAEA	OPHN1	RABGEF1	SCARNA13	TAF9B	UBE2Z
ABCB9	C1orf159	DCTN2	GATSL3	KCNJ9	MAGI2-AS3	ORAOV1	RAD17	SCEL-AS1	TAOK1	UBE4A
ABI2	C1QTNF9B	DCTN5	GBE1	KCNS1	MAN2A1	OSBPL1A	RAD50	SCFD1	TAOK3	UBR2
AC005550.5	C2	DDB2	GCGR	KDM8	MANSC1	OSBPL2	RAI14	SCGN	TARS	UBR5
AC007271.3	C21orf67	DDX3Y	GGA2	KIAA0195	MAOA	OSMR	RALGAPA2	SCLY	TAT	UCKL1
AC009158.1	C2CD3	DDX5	GHITM	KIAA0355	MAP2K2	OSTM1	RANBP2	SEC23A	TBC1D12	UFM1
AC010880.1	C3	DDX60L	GIGYF2	KIAA1033	MAP3K10	OTUD4	RARRES1	SEC23IP	TBC1D9	UGDH
AC093495.4	C5orf42	DGCR14	GK5	KIAA1109	MAP3K14	OTUD5	RASA4	SEC31A	TBL1XR1	UGGT2
AC096559.2	C7orf43	DGKQ	GLB1	KIAA1191	MAP3K6	OTUD7A	RB1	SEC63	TCOF1	UHRF2
ACAN	C9orf117	DHX9	GLG1	KIF1C	MAP4K3	OXCT1	RBBP9	SEL1L3	TCP11L1	ULK3
ACBD5	C9orf173	DICER1	GMFB	KIF20A	MAPK1	OXR1	RBCK1	SELT	TDRD9	ULK4P1
ACLY	C9orf3	DIRC2	GNAQ	KIF21A	MAPK10	PABPC1	RBL1	SEMA4D	TECPR1	UNC13A
ACO1	C9orf62	DKFZP434K028	GNN	KIF21B	MAPK1IP1L	PAIP1	RBM33	SENP1	TECR	UNC80
ACTG1	CA7	DLEU2	GNPTAB	KIF2A	MAPKAP1	PAK2	RBM39	SENP6	TENC1	UNK
ACVR1B	CACNA2D4	DLG1	GOLGA8A	KIF5B	MARCH2	PAK4	RCN1	SEPT10	TFDP1	UPB1
ACYP2	CADPS	DLG3	GPNMB	KIF6	MARCH6	PAM	REPS1	SEPT11	TFE3	URB1
ADAM23	CALM2	DLGAP1	GPR107	KIR2DS3	MARK4	PAN3	RFPL2	SEPT2	TGFBI	UROD
ADAM9	CALM3	DMXL2	GPR39	KLF7	MARS	PAPD4	RFX3	SETX	THADA	USMG5
ADAMTS12	CAMK2D	DNAH14	GPR97	KLHL20	MASP2	PAPD5	RFX7	SF3B1	THAP3	USP24
ADAMTSL1	CANX	DNAH17	GRAMD1B	KLHL23	MAST3	PAPOLA	RGL2	SH3BGRL	THAP5	USP34
ADD1	CAPN1	DNAH5	GRIK3	KLHL24	MATR3	PARK7	RGPD6	SH3BGRL2	THOC5	USP37
ADRM1	CAPRIN2	DNAJB4	GRM4	KLHL3	MBNL2	PARN	RGS14	SH3RF2	THSD4	USP3-AS1
AHI1	CARKD	DNAJC13	GRN	KMT2A	MBNL3	PARP4	RIF1	SHANK3	TIA1	USP4
AIM1L	CASC1	DNASE1L1	GRXCR1	KMT2E	MBTPS2	PCBP2	RNF123	SHF	TIAF1	USP43
AK9	CASP3	DNM1	GSK3B	KPNA2	MCAT	PCNX	RNF20	SIGLEC1	TIPRL	USP45
AKR1A1	CASP7	DNM1P47	GSR	KPNA5	MCOLN1	PCNXL2	RNF217	SIN3B	TLK1	USP9X
AKR1C1	CAST	DNMT1	GTF2I	KPTN	MED1	PCNXL4	RNGTT	SKIV2L2	TLL2	UVRAG
ALDH1L2	CAV2	DPM1	GTF2IP1	KRT10	MED13	PCSK5	ROBO1	SKOR1	TLN2	VAMP7
ALDH3A2	CBR3-AS1	DPP4	GUCA1A	KTN1	MED15	PCYOX1	ROCK1	SLAIN2	TM9SF4	VEPH1
AMMECR1	CBWD1	DPY19L3	GUCY2F	LAMA1	MED23	PDCD10	ROPN1	SLC16A1	TMED10	VIM
AMY1B	CBWD5	DPYD	GUSBP9	LAMA4	MEG9	PDCD2	RP11-11N9.4	SLC22A31	TMEFF1	VIPR2
ANAPC1	CBX3	DPYSL2	H2AFZ	LAMB4	MFSD11	PDCD6IP	RP11-15B24.5	SLC25A45	TMEM131	VMP1
ANAPC16	CBX7	DRAM1	HCG21	LAMTOR1	MGAM	PDE8A	RP11-174G17	SLC35A5	TMEM161B	VPS13A
ANK3	CCDC103	DSCAM	HDAC6	LARP1	MGMT	PDIA5	RP11-18B16.2	SLC35B1	TMEM177	VPS13C
ANKRD13B	CCDC132	DSE	HDAC9	LARP7	MICAL1	PDILT	RP11-196D18.1	SLC37A2	TMEM181	VPS41
ANKRD28	CCDC149	DSG2	HECTD4	LATS2	MICB	PDLIM2	RP11-216N14.8	SLC39A14	TMEM209	VTI1A
ANKRD36	CCDC18	DST	HELB	LBR	MIR1302-2	PDS5A	RP11-217E22.5	SLC41A3	TMEM234	VWA5A
ANKRD36B	CCDC37	DYNC1H1	HERC2	LCLAT1	MIR143HG	PER2	RP11-244K5.4	SLC6A8	TMEM243	VWA8
ANKS6	CCDC47	ECHDC1	HERC2P2	LDHA	MLYCD	PFKP	RP11-348F1.3	SLC9A9	TMEM30A	WDR3
ANXA5	CCDC50	EDRF1	HERC2P3	LDHB	MME	PHC3	RP11-436F23.1	SLC9C1	TMEM51	WDR44
ANXA6	CCND3	EHBP1	HHEX	LEPRE1	MMP2	PHF20L1	RP11-552M14.1	SLK	TMEM59	WDR46
ANXA8	CCNL1	EHBP1L1	HIF1A	LGALS1	MON1A	PHKB	RP11-767N15.1	SLMAP	TMEM87B	WDR6
ANXA8L2	CCPG1	EIF3E	HILS1	LGMN	MORF4L2	PI4K2B	RP11-782C8.5	SLTM	TMEM88B	WFDC3
AOC1	CCT3	EIF4E3	HIPK1	LGR4	MOSPD1	PIGT	RP4-613B23.1	SMARCA1	TMOD3	WHSC1L1
AP1S2	CCT6P3	EIF4EBP2	HK2	LILRA2	MPDZ	PIK3C3	RP5-1180C18.1	SMARCC1	TMPRSS15	WNT5A
AP3B1	CCZ1B	EIF5A	HLA-A	LINC00035	MPP6	PIK3CB	RP6-74O6.3	SMARCD3	TMPRSS6	WWP1
AP3M2	CD200R1	ELK3	HLA-DPB1	LINC00317	MPRIP	PIKFYVE	RPL24	SMC4	TMTC3	XPNPEP1
APCDD1L-AS1	CD38	ELMOD2	HLA-DRA	LINC00461	MRPL3	PITRM1	RPL3	SMC6	TNC	XPO1
APP	CD44	ELOVL5	HLA-DRB1	LINC00559	MSH3	PITX2	RPL31	SMCHD1	TNFRSF13B	XRCC3
APPBP2	CD58	ENPP1	HLA-DRB3	LINC00593	MSI1	PIWIL3	RPL32	SMIM24	TNFRSF14	XRCC5
APPL2	CDC42BPA	ENPP6	HLA-H	LINC00636	MSMO1	PKD1	RPL38	SMURF1	TNKS2	YIPF6
ARFGEF2	CDC5L	EPAS1	HMCN1	LINC00649	MTA3	PKD2	RPL41	SNORA71E	TNN	YME1L1
ARHGAP18	CDC73	EPHA6	HMCN2	LINC00893	MTAP	PKHD1	RPN2	SNORD50A	TNNC2	ZBED1
ARHGAP26	CDCA5	EPHB6	HNRNPA1P10	LINC00959	MTHFD1	PKIA	RPS13	SNX13	TNNT2	ZBTB22
ARHGAP39	CDCA7	EPT1	HNRNPK	LIPA	MTHFD2	PLA2G2A	RRH	SNX29	TNNT3	ZBTB38
ARHGAP8	CDH1	EPX	HNRNPL	LITAF	MTMR2	PLA2G4E	RRM1	SNX3	TNXB	ZDHHC20
ARHGEF11	CDH22	ERBB2IP	HNRNPUL1	LLGL1	MTMR7	PLAA	RRN3	SORBS2	TOLLIP	ZEB1
ARHGEF7	CDK11A	ERCC6	HOXD10	LMCD1-AS1	MYH10	PLAT	RSBN1L	SPAG1	TOP1	ZFAND6
ARID2	CDK12	ERGIC3	HSD17B7	LOC100132891	MYH16	PLAU	RSRC2	SPARC	TOP2B	ZFC3H1
ARIH2	CDK19	ERICH6	HSP90B1	LOC100422737	MYH3	PLCD1	RSRP1	SPARCL1	TOR1AIP1	ZFP64
ARL2BP	CDK4	ERLIN2	HSPA1B	LOC100996291	MYLK	PLCE1	RTTN	SPATA21	TOX	ZFYVE1
ARMC8	CDON	ESRP2	HSPA6	LOC101060483	MYO10	PLOD2	RUNX1T1	SPATA31A5	TPPP3	ZHX3
ARVCF	CEL	ETFA	HSPA8	LOC101927641	MYO15A	PLP1	RYR2	SPCS3	TPR	ZIC1
ASMTL-AS1	CELSR2	EXOSC10	HSPB1	LOC101927768	MYO15B	PLS3		SPEF2	TRAF3IP2	ZNF131
ASXL2	CEP104	EXT2	HSPB11	LOC101927843	MYO3B	PMP22		SPIDR	TRAM1	ZNF215
ATAD2B	CEP112	EXTL2	HSPG2	LOC101927902	MYO9B	PMVK		SPIRE1	TRAPPC8	ZNF22
ATAD3A	CEP63	FABP3	HUWE1	LOC101928495	MYT1L	PNCK		SPOPL	TRIM24	ZNF227
ATAD3C	CERS2	FADS1	IDH3B	LOC101928782	NAA15	PNISR		SPRYD7	TROVE2	ZNF23
ATG2B	CERS4	FADS2	IFRD1	LOC101928978	NAA40	PNMA2		SPTBN5	TRPS1	ZNF274
ATIC	CFC1B	FAM114A1	IFRD2	LOC101929653	NAMPT	PNPLA8		SQLE	TRPV2	ZNF280C
ATM	CFDP1	FAM118A	IFT122	LOC101930595	NBEAL1	POC1B		SQSTM1	TSHZ2	ZNF283
ATMIN	CHD2	FAM13B	IGHG2	LOC283299	NBEAL2	POLL		SRBD1	TSPO2	ZNF331
ATP1A1-AS1	CHDH	FAM13C	IGHMBP2	LOC340515	NBN	POM121C		SRCIN1	TSTA3	ZNF365
ATP1B3	CHM	FAM179B	IKBKAP	LOC644961	NBPF9	PORCN		SREK1	TTC13	ZNF398
ATP1B4	CHMP5	FAM199X	IKBKB	LOC645202	NCL	POU4F3		SRP14	TTC17	ZNF492
ATP6V1B2	CHN1	FAM204A	IKZF2	LOC645355	NCOA7	PPIP5K2		SRP72	TTC28	ZNF503-AS1
ATP6V1H	CHRD	FAM208A	IL10RB	LOC645513	NCOR1	PPP1CB		SRP9	TTC3	ZNF529
ATRN	CIDECP	FAM86C2P	IL17RE	LOC650368	NCOR2	PPP1R13B		SRPX	TTLL5	ZNF555
ATRX	CKAP2L	FAM89B	IL6R	LRBA	NCSTN	PPP2R3C		SRPX2	TUBA1A	ZNF672
ATXN3	CLASP2	FAM98A	IL6ST	LRP2	NDEL1	PPP2R5C		SRR	TUBA1B	ZNF93
ATXN7L2	CLASRP	FAP	IL9R	LRPPRC	NDRG3	PPP2R5D		SRRT	TXNDC11	ZNRD1
AWAT2	CLCN5	FARSB	ILK	LRRC1	NDST2	PPP3CC		STAG1	TXNIP	ZSWIM8
B2M	CLIP2	FAS	INTS3	LRRC28	NDUFA3	PPP4R1		STAG2	TXNL1	ZZEF1
B3GAT1	CLN6	FASN	INTS6	LRRC37A	NDUFA5	PRCC		STEAP2		
B3GNT2	CLU	FASTK	IPO5	LRRC37BP1	NDUFA8	PRDM13		STEAP4		
B3GNT5	CNBD1	FAT2	ITCH	LRRC41	NDUFB8	PRKAA1		STIL		
BACH1	CNOT6	FBXL3	ITFG1	LRRC45	NEK1	PRKAG1		STK10		
BARHL1	CNPY2	FBXL5	ITGAM	LRRFIP1	NELL1	PRKAR1B		STK3		
BBS4	COG6	FBXO27	ITGAV	LRRK2	NETO2	PRKAR2A		STK39		
BBS7	COL11A1	FBXO33	ITGB1BP1	LSM14A	NF1	PRKCI		STMN3		
BBS9	COL11A2	FBXO38	ITGBL1	LTBP1	NFE4	PRKD3		STPG1		
BCAP31	COL12A1	FBXW11	ITIH5	LTBP2	NFRKB	PRKDC		STX2		
BCKDHB	COL14A1	FCGR2C	ITPR2	LUC7L3	NFX1	PRKG2		STXBP2		
BCL7C	COL15A1	FCHO2	ITPR3	LY9	NGB	PRKY		STXBP5		
BCOR	COL18A1	FER1L4	JUP	LYRM1	NIPA2	PROM1		SUCO		
BIRC6	COL18A1-AS2	FERMT2		LYST	NIPSNAP1	PROS1		SUFU		
B2M	COL22A1	FKBP15			NKAIN4	PRPS2		SULF1		
B3GAT1	COL4A2	FKBP7			NLRP12	PRRC2C		SUN2		
B3GNT2	COL4A5	FLII			NME1	PSAP		SVOP		
B3GNT5	COL5A1	FN1			NNT	PSMA7		SYNJ2		
BACH1	COL9A2	FOXJ3			NOL8	PSMB10		SYT1		
BARHL1	COPB1	FOXP2			NONO	PSMB5		SYTL3		
BBS4	COPG2	FRAS1			NOP58	PSMD13				
BBS7	COPS2	FRMPD2			NOX5	PTCH1				
BBS9	COPS7A	FSCN3			NPIPA1	PTK2				
BCAP31	CORO1C	FXR1			NPM1	PTK2B				
BCKDHB	CPB2-AS1				NR2F1-AS1	PTMA				
BCL7C	CPEB2				NRAS	PTP4A2				
BCOR	CPNE3				NRD1	PTPLA				
BIRC6	CPSF2				NT5C	PTPLB				
BLM	CPSF6				NT5DC2	PTPN1				
BMS1	CPSF7				NTNG1	PTPN11				
BNIP1	CPT1B				NUB1	PTPN14				
BOD1L1	CREB1				NUCB2	PTPRA				
BOLA3	CREB3L2				NUDCD1	PTPRK				
BOP1	CRELD1				NUSAP1	PTPRM				
BPI	CROCCP2					PTPRN				
BPTF	CSNK1G3					PTPRQ				
BRCA2	CSTF3					PTRF				
BRD7	CTA-342B11.2					PUM1				
BTBD1	CTB-174D11.2					PUM2				
BTN1A1	CTD-2335O3.3					PUS3				
BZW1	CTNNB1					PUSL1				
	CTSF					PWP2				
	CTU2					QARS				
	CWC22									
	CYB5D1									
	CYC1									
	CYFIP1									
	CYP27A1									

Table S4: RIDD regulated genes (intersection of the list in S4 and genes upregulated in IRE1 DN cells)

**RIDD**
ADAM9
ANXA5
ARHGAP18
ATG2B
CALM1
CALM2
CALM3
CAMK2D
CASP7
CAV2
CDC42BPA
CDC5L
COPB2
DHX9
DST
ENPP1
FBXO38
FERMT2
HSPA1A
HSPA1B
IL1R1
INTS6
ITGAV
KIF20A
KLF7
LARP7
LGR4
PRKD3
RBBP9
SPEF2
TMOD3
TNC
TPR
TROVE2
TTC37
UBE2D3
ZNF22

Table S5: List of potential miR-17 regulated genes, in red are those exhibiting a miR-17 predicted binding site.

**miR17 sign**
BGLAP
CD59
CELSR2
COL13A1
DHRS2
DIRAS3
DKK3
EFEMP1
EXOSC6
FGF14
FOXF1
GAP43
GPR177
GPR37
IGFBP2
KIAA0746
KRTAP1-5
LIFR
LMO2
LPPR4
MAGEC1
MAN1C1
NDP
NOS1
NR2F1
PCDH17
PPL
PROS1
SHROOM3
SLC14A1
SLC1A1
TMEFF2
TNXA
TSLP
ZNF138
ZNF20
ZNF738
ZNF85

Table S6: Primers used for site-directed mutagenesis

Mutation (AA)	Sens	Primer sequence (5'-3')
S769F	FWD	GCGTCTTTTACTACGTAATCTTTGAGGGCAGCCACCCTTTTGGC
	REV	GCCAAAAGGGTGGCTGCCCTCAAAGATTACGTAGTAAAAGACGC
Q780*	FWD	CCCTTTTGGCAAGTCCCTGTAGCGGCAGGCCAACATCC
	REV	GGATGTTGGCCTGCCGCTACAGGGACTTGCCAAAAGGG
P336L	FWD	ACAAGGGGGAGTGTGTGATCACGCTCAGCACGGACGTCAA
	REV	TTGACGTCCGTCCTGAGCGTGATCACACACTCCCCCTTGT
A414T	FWD	CCAGACTTCAGAAAACACACCTACCACCGTGTCTCGGGA
	REV	TCCCGAGACACGGTGGTAGGTGTGTTTTCTGAAGTCTGG

Table S7: RT-qPCR primers used in this study

	Sense	Primer sequence (5'-3')
BiP	FWD	GCTTATGGCCTGGATAAGAGG
	REV	CCACAACTTCGAAGACACCAT
Chop	FWD	ATTGACCGAATGGTGAATCTGC
	REV	AGCTGAGACCTTTCCTTTTGTCTA
Col6A1	FWD	CCCTCGTGGACAAAGTCAAG
	REV	GTTTCGGTCACAGCGGTAGT
Edem	FWD	AGTCATCAACTCCAGCTGGAA
	REV	AACCATCTGGTCAATCTGTCG
Erdj4	FWD	TGGTGGTTCCAGTAGACAAAGG
	REV	CTTCGTTGAGTGACAGTCCTGC
Gadd34	FWD	CCTCTACTTCTGCCTTGTCTCCAG
	REV	TTTTCCTCCTTCTTCTCGGACG
Grp94	FWD	TCCTCCTCCTGACGTTGTAAA
	REV	TGCTCGCCATCTAGTACATCC
Herpud	FWD	CTATTCCGCCTTCCTTGTAGC
	REV	CCTCTTGGGTCAGCAATTACA
Orp150	FWD	GAAGATGCAGAGCCCATTTC
	REV	TCTGCTCCAGGACCTCCTAA
Pdgfrb	FWD	TCCATCCCTCTGTTCTCCTG
	REV	CTGCCCTCTCCCAGTTATCA
Per1	FWD	TATACCCTGGAGGAGCTGGA
	REV	AGGAAGGAGACAGCCACTGA
Scara3	FWD	CGCTGCCAGAAGAACCTATC
	REV	AACCAGAGAGGCCAACACAG
Sparc	FWD	GGCCTGGATCTTCTTTCTCC
	REV	CCACCACCTCTGTCTCATCA
Actin	FWD	CATGGGTGGAATCATAATGG
	REV	AGCACTGTGTTGCGCTACAG
Gapdh	FWD	AAGGTGAAGGTCGGAGTCAA
	REV	CATGGGTGGAATCATAATGG
FOXF1	FWD	CCCAGCATGTGTGACCGAAA
	REV	ATCACGCAAGGCTTGATGTCT
TSLP	FWD	TGCCTTAGCTATCTGGTGCC
	REV	ACGCAACAATCCTTGTAATTG
PTEN	FWD	AGGGACGAACTGGTGTAATGA
	REV	CTGGTCCTTACTTCCCCATAGAA
MAN1C1	FWD	CGATACCCTCTACCTCATGGAG
	REV	CGCTCACGTTCAGGTGGAA
LMO2	FWD	TCTGCCGGAGAGACTATCTCA
	REV	ATAGGCACGAATCCGCTTGTC
TGFB1	FWD	GTCAATGTACAGCTGCCGCA
	REV	GTCAATGTACAGCTGCCGCA
VIM	FWD	GACGCCATCAACAACGAGTT
	REV	CTTTGTCGTTGGTTAGCTGGT
ZEB1	FWD	GATGATGAATGCGAGTCAGATGC
	REV	ACAGCAGTGTCTTGTTGTTGT
VEGFA	FWD	CGAACCCATGAACTTTCTGC
	REV	CCTCAGTGGGCACACACTCC
XBP1s	FWD	TGCTGAGTCCGCAGCAGGTG

	REV	GCTGGCAGGCTCTGGGAAAG
XBP1tot	FWD	CCTGGTTCTCAACTACAAGGC
	REV	AGTAGCAGCTCAGACTGCCA
MMP-9	FWD	ACCTCGAACTTTGACAGCGAC
	REV	GAGGAATGATCTAAGCCCAGC
IRE1	FWD	GCCACCCTGCAAGAGTATGT
	REV	ATGTTGAGGGAGTGGAGGTG
CXCL2	FWD	CTGCGCTGCCAGTGCTT
	REV	CCTTCACACTTTGGATGTTCTTGA
CCL2	FWD	CAAGCAGAAGTG GGTTCAGGAT
	REV	TCTTCGGAGTTTGGGTTTGC
IL6	FWD	GGTACATCCTCGACGGCATCT
	REV	GTGCCTCTTTGCTGCTTTCAC
IL8	FWD	TGGCAGCCTTCCTGATTTCT
	REV	GGGTGGAAAGGTTTGGAGTATG

Table S8: antibodies used in this study.

Antigen	Usage	Dilution	Company	Reference
CANX	WB	1/1000	N/A	Chevet et al. EMBOJ (1999)
XBP1s	IHC	1/200	N/A	Lhomond et al. Meth. Mol. Biol. (2015)
VIM	IHC	1/250	DAKO	M0-725 clone B9 11300
IBA1	IHC	1/050	WAKO	019-19741
CD31	IHC	1/050	DIANOVA	DIA3105231
IRE1	WB	1/1000	SANTACRUZ	H-190
PDGFRB	WB	1/1000	SANTACRUZ	958
P53	WB	1/1000	ABCAM	DO-1
ACTIN	WB	1/1000	ABCAM	ab8227
CD11b	FACS	N/A	BD	ICRF44
CD45	FACS	N/A	BD	2D1
KDEL	IF	1/250	ABCAM	ab12223
CD31	FACS	N/A	BD	WM59

A) *Supplementary Figure legends*

Figure S1: Bioinformatics workflow for the identification of 38-hub genes representative of the IRE1 signature. Raw data (*.CEL files) from the GSE27306 dataset (Pluquet et al, 2013) were processed into R/Bioconductor by using the RMA normalization and Limma package, and 1051 differentially expressed (D.E.) genes were selected between DN and WT U87 cells by using a corrected P value (pval) threshold of 0.05 and fold change threshold of $|\log2(F.C.)| \geq 1.5$. D.E. gene list was then introduced into the BioInfoMiner tool (see suppl. materials and methods) and gene prioritization was executed based on the biomedical ontologies of four different functional and phenotype databases including GO, Reactome, MGI and HPO, separately. For the annotation process was used the "complete" version (see materials and methods) and the hypergeometric pvalue threshold was set to 0.05. Two-hundred twenty-seven (227) highly prioritized genes including their proximal interactors corresponded to the union of the BioInfoMiner output from the four databases and 38 hub-genes were highlighted as the intersection with the IRE1 signature of (Pluquet et al, 2013).

Figure S2: IRE1 signaling signature in the GBMmark cohort - A. Hierarchical clustering of GBM patients (GBMmark cohort) based on high or low IRE1 activity as assessed with the expression of the IRE1 gene expression signature. **B.** Expression of microglial/monocyte/macrophage markers mRNA IBA1 in the IRE1high (red) and IRE1low (green) populations. **C.** Expression of angiogenesis marker mRNA CD31 in the IRE1high (red) and IRE1low (green) populations. **D.** Expression of the migration marker mRNA RHOA in the IRE1high (red) and IRE1low (green) populations. **E.** Gene expression analysis in the GBMmark cohort tumors exhibiting high (red) or low (green) IRE1 activity for targets of the three UPR arms: CHOP (PERK), ERDJ4, EDEM1 (IRE1) and ERO1LB (ATF6).

Figure S3: Identification of somatic mutations in IRE1 in GBM. A. Specific IRE1 exons sequencing flowchart: DNA was extracted from 23 gliomas samples provided by the Bordeaux Tumor Bank and IRE1alpha exons sequences were compared to normal brain tissue IRE1alpha sequence. One of the 23 samples showed a novel IRE1alpha mutation, as indicated by the red arrow on DNA sequence representation. **B.** Tumor characterization from the 70 years-old female patient that presented the A414T mutation. Immunohistochemistry staining revealed a mesenchymal-like encapsulated tumor (Hematoxylin and eosin stain: HES), highly vascularized as indicated by CD31 staining of endothelial cells. IRE1alpha activation within the tumor is visible by overexpression of the spliced form of XBP1 (XBP1s) in the tumoral tissue (T) compared to the non-tumoral tissue (NT). **C.** Sequence alignment of

IRE1 proteins reveals that Pro^{336} and Ser^{769}residues but not Ala^{414} are well conserved in IRE1 proteins. Numbers refer to residue positions in human IRE1 protein (ERN1); *D. melanogaster*; *C. elegans*; *S. cerevisiae*. **D.** Three-dimensional reconstruction of human IRE1 domains (luminal and cytosolic) based on existing structures and positioning of the different mutations (those found in GBM are boxed in red).

Figure S4: Functional analysis of IRE1 variants in U87 cells. A. U87 were transduced with empty pCDH lentivector (EV) or with pCDH lentivector containing the WT (WT) or the mutated (S769F, Q780*, P336L, A414T) IRE1alpha coding sequence and evaluated by immunoblot (anti-IRE1alpha and anti-actin). This revealed a 10-fold overexpression of full length (100kDa) IRE1alpha protein in WT, S769F, P336L and A414T conditions and over expression of a truncated (80kDa) IRE1alpha protein in Q780* condition. **B.** Confocal immunofluorescence studies performed with U87 cells expressing these variant proteins showed co-localization of WT or mutated IRE1alpha (red) with the ER marker anti- KDEL (green). **C.** DSP-mediated in vivo cross-linking of IRE1 proteins in mutant expressing U87 cells. Anti-IRE1alpha immunoblot under both reduced (top) and non-reduced (bottom) revealed IRE1alpha oligomerization in basal conditions due to over expression of WT, P336L and A414T but not S769F nor Q780* IRE1alpha variant proteins. **D.** EtBr-stained agarose gel of XBP1 cDNA amplicons corresponding to unspliced (XBP1u) and spliced (XBP1s) forms of XBP1 mRNA revealed XBP1 splicing in basal conditions or upon tunicamycin treatment (TUN, 5µg/mL for 6 hrs). **E.** The expression levels of miR-17-5p were quantified in IRE1 DN cells and IRE1 P336L expressing U87 cells exposed or not to the IRE1 RNase inhibitor MKC4485. Data are means ±SD. **F.** Expression of miR-17-5p in U87 cells expressing IRE1 variants under basal conditions (grey bars) or tunicamycin-induced stress (white bars). Data are the mean ±SD of 4 independent experiments.

Figure S5: Phenotypic impact of the IRE1 variants in U87 cells. A. Bar graph representing the doubling time of U87 population for each condition. **B.** Representative imaging of the sphere phenotypes at 6 hrs and 48 hrs post-seeding in agar coated well of a 96-well plate. The curve representation of the neurosphere size along 48 hrs did not reveal any significant differences in terms of cell aggregation and adhesion. Bar graphs represent the sphere size at 6 hrs post-seeding (top) and of the rate of sphere formation (bottom). All data shown are mean ± SEM of at least three biological replicates. **C.** Analysis of the KEGG pathway for glioma and annotated for component previously identified to be regulated (expression or activity) by IRE1 (yellow). **D.** Basal expression of PDGFRbeta and p53 in those experiments protein expression was standardized with Calnexin (CANX). **E.** Analysis

of p53 mRNA expression in U87 control (Empty vector – EV) or in cells expressing the IRE1 P336L variant.

Figure S6: XBP1s in GBM tumors. A. Heat map representation and functional clustering of GBM patients of the GBMmark cohort based on the XBP1s signature (**Table S2**). **B.** Immunohistochemical analysis of 24 GBM tumor sections using anti-XBP1s (blue) or anti IBA1 (black) antibodies.

Figure S7: **A.** Approach for identifying RNA substrates cleaved by IRE1 in vitro. **B.** Intersection of the list of RNA cleaved by IRE1 *in vitro* (**Table S4**) and that of mRNA whose expression is upregulated in IRE1 DN cells under basal conditions. **C.** Relative distribution of the different classes of GBM - proneural (blue), neural (orange), classical (green), and mesenchymal (red) according to the tumor status, namely XBP1+/RIDD-; XBP1+/RIDD+; XBP1-/RIDD+; XBP1-/RIDD-. **D**. Correlation between the XBP1+ tumor groups in the TCGA RNAseq cohort and the presence of identified reads corresponding to the spliced XBP1. **E.** Kaplan-Meier survival curves of $XBP1s^{high}/RIDD^{low}$ (red), $XBP1s^{high}/RIDD^{high}$ (grey), $XBP1s^{low}/RIDD^{low}$ (blue) and $XBP1s^{low}/RIDD^{high}$ GBM tumor patients of both TCGA cohorts (microarrays (left) and RNAseq (right)).

Figure S8: Characterization of the IRE1-modified primary GBM lines. A. Expression levels (as determined using RT-qPCR; n = 3 per clone) of IRE1 mRNA in parental RNS85, 87, 96 and 130 and in the same lines overexpressing IRE1 WT or IRE1 Q780*. **B.** Phase contrast pictures of the primary lines expressing or not IRE1 WT or IRE1 Q780*.

B) Supplementary References

Ashburner M, Ball CA, Blake JA, Botstein D, Butler H, Cherry JM, Davis AP, Dolinski K, Dwight SS, Eppig JT et al (2000) Gene ontology: tool for the unification of biology. The Gene Ontology Consortium. Nat Genet 25**:** 25-29

Chatziioannou AaM, P. (2011) Exploiting statistical methodologies and controlled vocabularies for prioritized functional analysis of genomic experiments: the StRAnGER web application. Front Neurosci 5**:** 8

Consortium EP, Birney E, Stamatoyannopoulos JA, Dutta A, Guigó R, Gingeras TR, Margulies EH, Weng Z, Snyder M, Dermitzakis ET et al (2007) Identification and analysis of functional elements in 1% of the human genome by the ENCODE pilot project. Nature 447**:** 799-816

consortium T (2008) Comprehensive genomic characterization defines human glioblastoma genes and core pathways. Nature 455**:** 1061-1068

Croft D, Mundo AF, Haw R, Milacic M, Weiser J, Wu G, Caudy M, Garapati P, Gillespie M, Kamdar MR et al (2013) The Reactome pathway knowledgebase. Nucleic Acids Res 42**:** D472-477

Dejeans N, Pluquet O, Lhomond S, Grise F, Bouchecareilh M, Juin A, Meynard-Cadars M, Bidaud-Meynard A, Gentil C, Moreau V et al (2012) Autocrine control of glioma cells adhesion and migration through IRE1alpha-mediated cleavage of SPARC mRNA. J Cell Sci 125**:** 4278-4287

Eppig JT, Richardson JE, Kadin JA, Ringwald M, Blake JA, Bult CJ (2015) Mouse Genome Informatics (MGI): reflecting on 25 years. Mamm Genome 26**:** 272-284

Kohler S, Doelken SC, Mungall CJ, Bauer S, Firth HV, Bailleul-Forestier I, Black GC, Brown DL, Brudno M, Campbell J et al (2013) The Human Phenotype Ontology project: linking molecular biology and disease through phenotype data. Nucleic Acids Res 42**:** D966-974

Moutselos K, Maglogiannis I, Chatziioannou A (2010) Delineation and interpretation of gene networks towards their effect in cellular physiology- a reverse engineering approach for the identification of critical molecular players, through the use of ontologies. Conf Proc IEEE Eng Med Biol Soc (EMBC) 2010**:** 6709-6712

Moutselos K, Maglogiannis I, Chatziioannou A (2011) GOrevenge: A novel generic reverse engineering method for the identification of critical molecular players, through the use of ontologies. . IEEE Trans Biomed Eng

Pilalis E, Chatziioannou A (2013) Prioritized functional analysis of biological experiments using resampling and noise control methodologies. IEEE 13th International Conference on " Bioinformatics and Bioengineering (BIBE)**:** 10-13

Pluquet O, Dejeans N, Bouchecareilh M, Lhomond S, Pineau R, Higa A, Delugin M, Combe C, Loriot S, Cubel G et al (2013) Posttranscriptional regulation of PER1 underlies the oncogenic function of IREalpha. Cancer Res 73**:** 4732-4743

Rempel SA (2001) Molecular biology of nervous system tumors. Hematol Oncol Clin North Am 15**:** 979-1006

Ritchie ME, Phipson B, Wu D, Hu Y, Law CW, Shi W, Smyth GK (2015) limma powers differential expression analyses for RNA-sequencing and microarray studies. Nucleic Acids Research 43: e47

Vichai V, Kirtikara K (2006) Sulforhodamine B colorimetric assay for cytotoxicity screening. Nat Protoc 1: 1112-1116

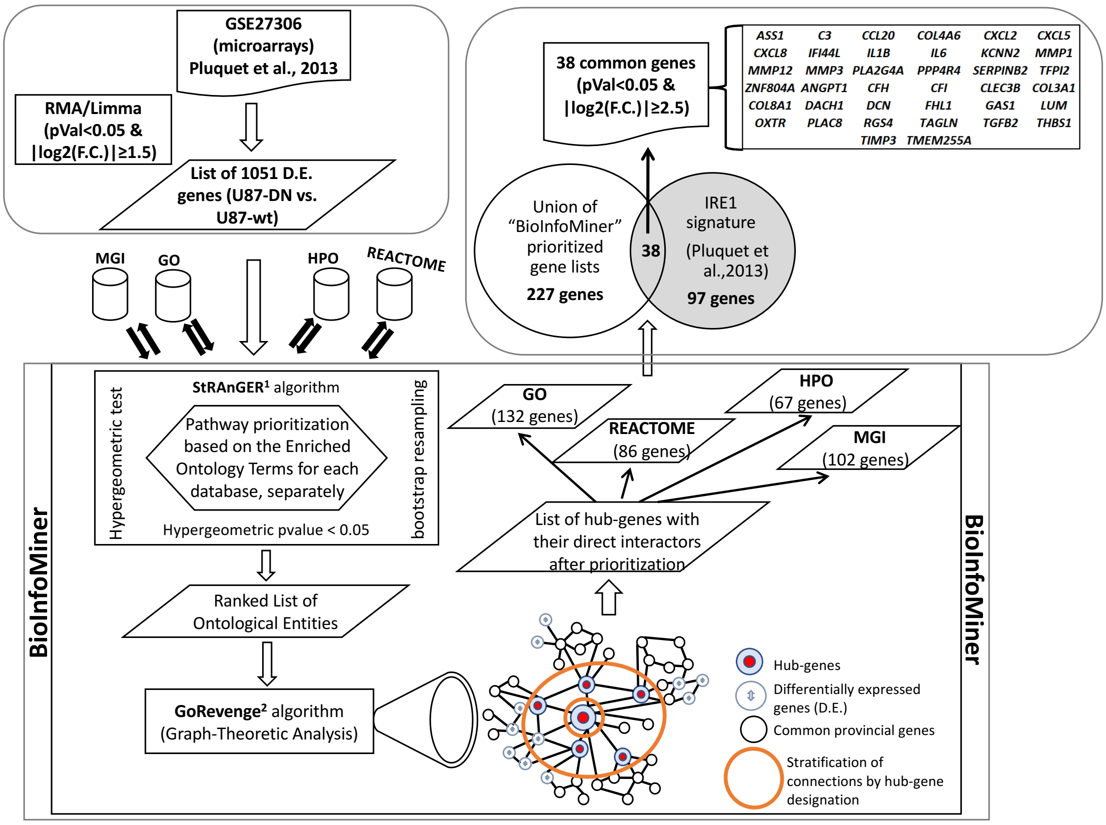

GSE27306
(microarrays)
Pluquet et al., 2013
RMA/Limma
(pVal<0.05 &
|log2(F.C.)|≥1.5)
List of 1051 D.E.
genes (U87-DN vs.
U87-wt)
MGI
GO
HPO
REACTOME
38 common genes
(pVal<0.05 &
|log2(F.C.)|≥2.5)
ASS1 C3 CCL20 COL4A6 CXCL2 CXCL5
CXCL8 IFI44L IL1B IL6 KCNN2 MMP1
MMP12 MMP3 PLA2G4A PPP4R4 SERPINB2 TFPI2
ZNF804A ANGPT1 CFH CFI CLEC3B COL3A1
COL8A1 DACH1 DCN FHL1 GAS1 LUM
OXTR PLAC8 RGS4 TAGLN TGFB2 THBS1
TIMP3 TMEM255A
Union of
"BioInfoMiner"
prioritized
gene lists
227 genes
38
IRE1
signature
(Pluquet et
al.,2013)
97 genes
BioInfoMiner
StRAnGER[1] algorithm
Hypergeometric test
Pathway prioritization
based on the Enriched
Ontology Terms for each
database, separately
bootstrap resampling
Hypergeometric pvalue < 0.05
Ranked List of
Ontological Entities
GoRevenge[2] algorithm
(Graph-Theoretic Analysis)
GO
(132 genes)
REACTOME
(86 genes)
HPO
(67 genes)
MGI
(102 genes)
List of hub-genes with
their direct interactors
after prioritization
Hub-genes
Differentially expressed
genes (D.E.)
Common provincial genes
Stratification of
connections by hub-gene
designation
BioInfoMiner

Figure S1

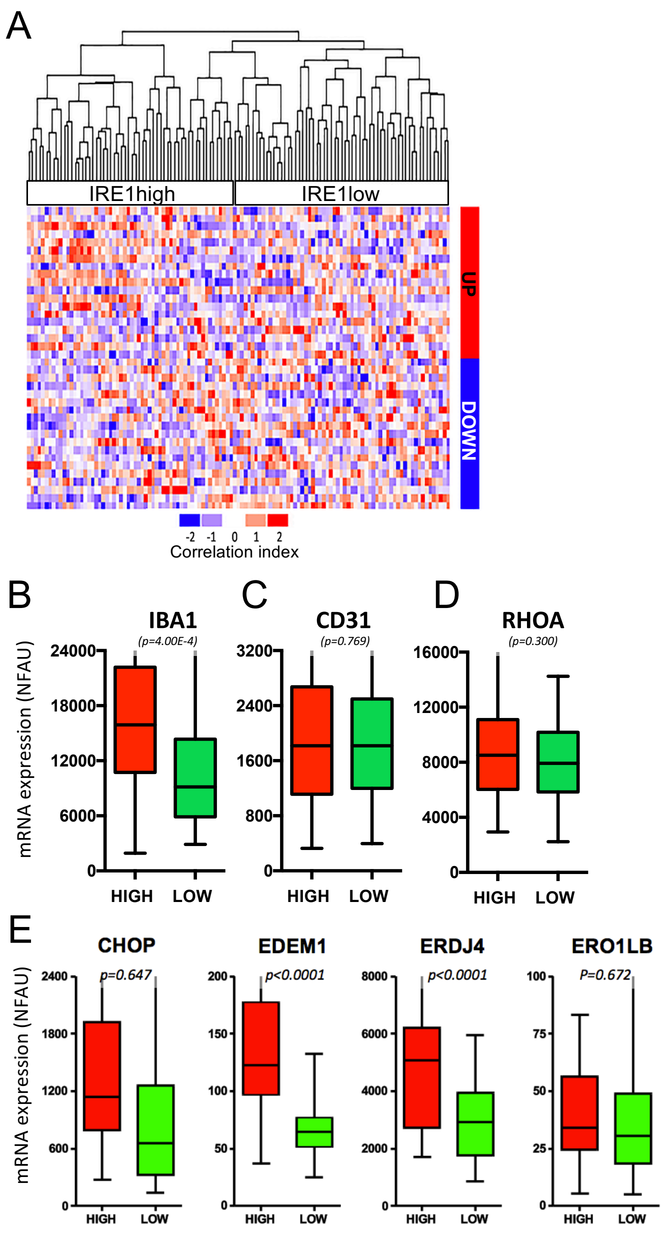
A
IRE1high
IRE1low
UP
DOWN
-2 -1 0 1 2
Correlation index
B
C
D
IBA1
(p=4.00E-4)
CD31
(p=0.769)
RHOA
(p=0.300)
mRNA expression (NFAU)
HIGH
LOW
E
CHOP
p=0.647
EDEM1
p<0.0001
ERDJ4
p<0.0001
ERO1LB
P=0.672

Figure S2

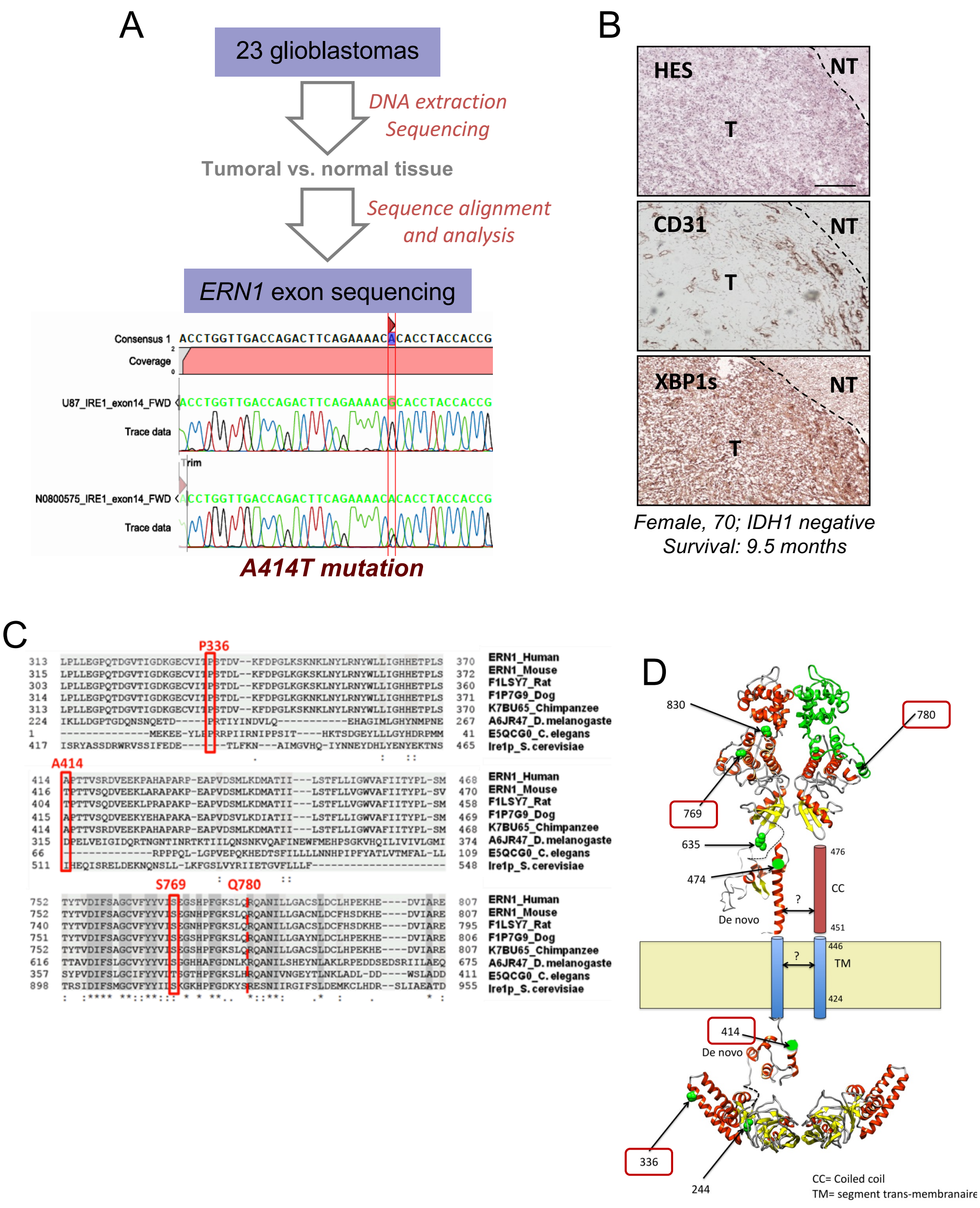
A
23 glioblastomas
DNA extraction
Sequencing
Tumoral vs. normal tissue
Sequence alignment
and analysis
ERN1 exon sequencing
Consensus 1
Coverage
U87_IRE1_exon14_FWD
Trace data
Trim
N0800575_IRE1_exon14_FWD
Trace data
A414T mutation
B
HES
NT
T
CD31
NT
T
XBP1s
NT
T
Female, 70; IDH1 negative
Survival: 9.5 months
C
P336
A414
S769
Q780
ERN1_Human
ERN1_Mouse
F1LSY7_Rat
F1P7G9_Dog
K7BU65_Chimpanzee
A6JR47_D.melanogaste
E5QCG0_C.elegans
Ire1p_S.cerevisiae
D
830
780
769
635
474
De novo
476
CC
451
446
TM
424
414
De novo
336
244
CC= Coiled coil
TM= segment trans-membranaire

Figure S3

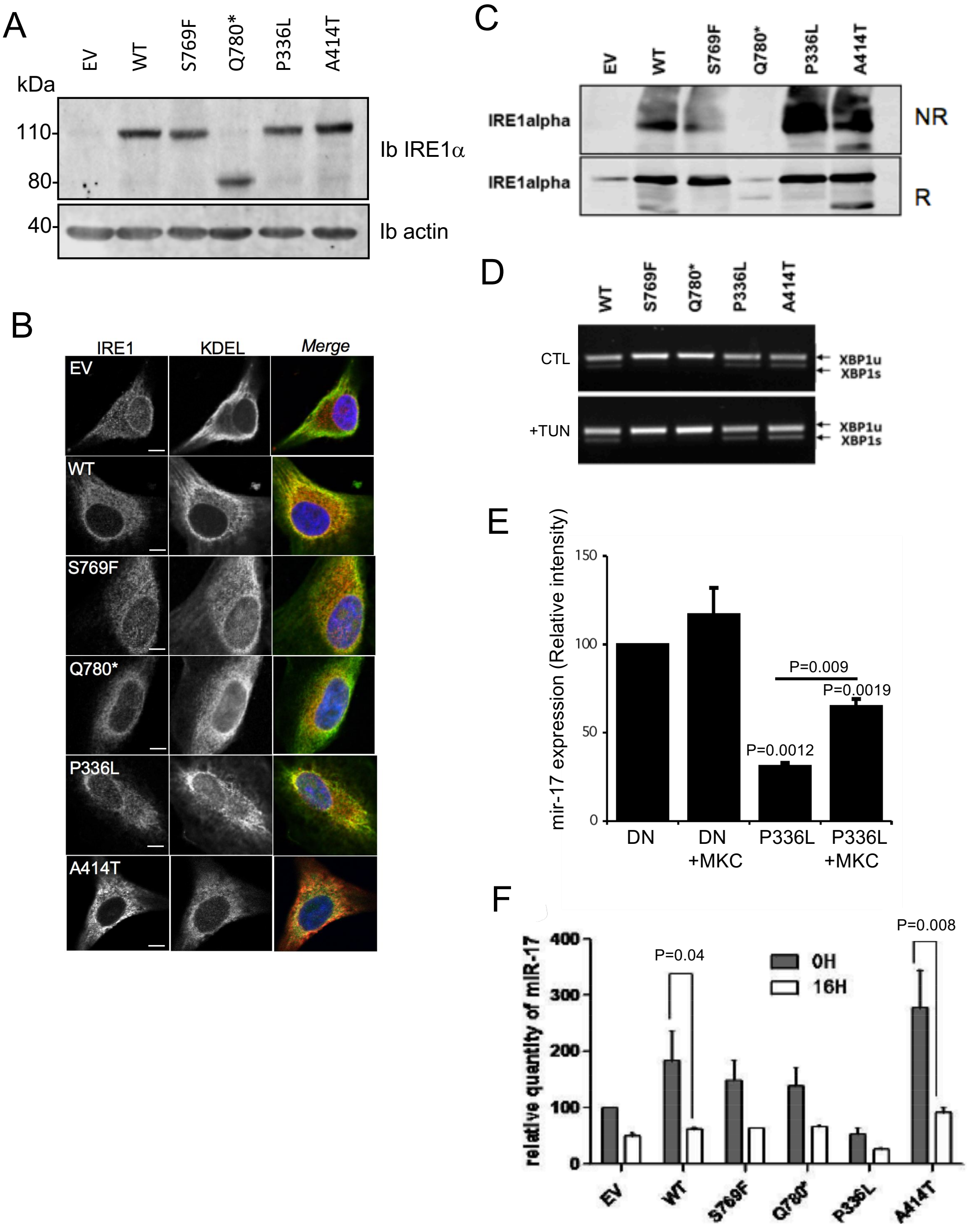
A
EV
WT
S769F
Q780*
P336L
A414T
kDa
110
80
40
Ib IRE1α
Ib actin
B
IRE1
KDEL
Merge
EV
WT
S769F
Q780*
P336L
A414T
C
EV
WT
S769F
Q780*
P336L
A414T
IRE1alpha
IRE1alpha
NR
R
D
WT
S769F
Q780*
P336L
A414T
CTL
+TUN
XBP1u
XBP1s
XBP1u
XBP1s
E
mir-17 expression (Relative intensity)
150
100
50
0
P=0.009
P=0.0019
P=0.0012
DN
DN +MKC
P336L
P336L +MKC
F
relative quantity of miR-17
400
300
200
100
0
P=0.04
P=0.008
0H
16H
EV
WT
S769F
Q780*
P336L
A414T

Figure S4

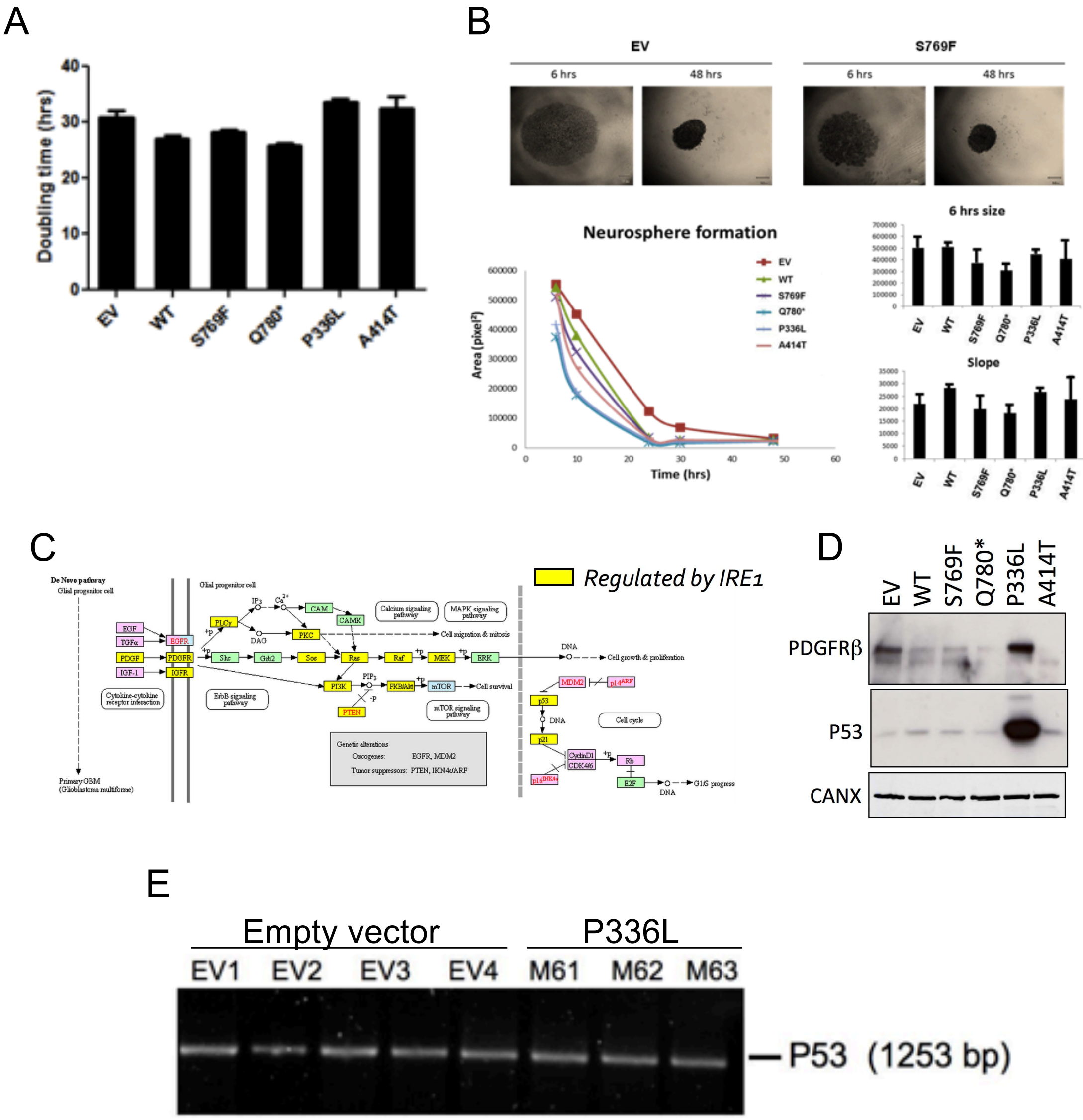
A
Doubling time (hrs)
40
30
20
10
0
EV
WT
S769F
Q780*
P336L
A414T
B
EV
S769F
6 hrs
48 hrs
6 hrs
48 hrs
Neurosphere formation
Area (pixel²)
600000
500000
400000
300000
200000
100000
0
0
10
20
30
40
50
60
Time (hrs)
EV
WT
S769F
Q780*
P336L
A414T
6 hrs size
Slope
C
Regulated by IRE1
D
EV
WT
S769F
Q780*
P336L
A414T
PDGFRβ
P53
CANX
E
Empty vector
P336L
EV1
EV2
EV3
EV4
M61
M62
M63
P53 (1253 bp)

Figure S5

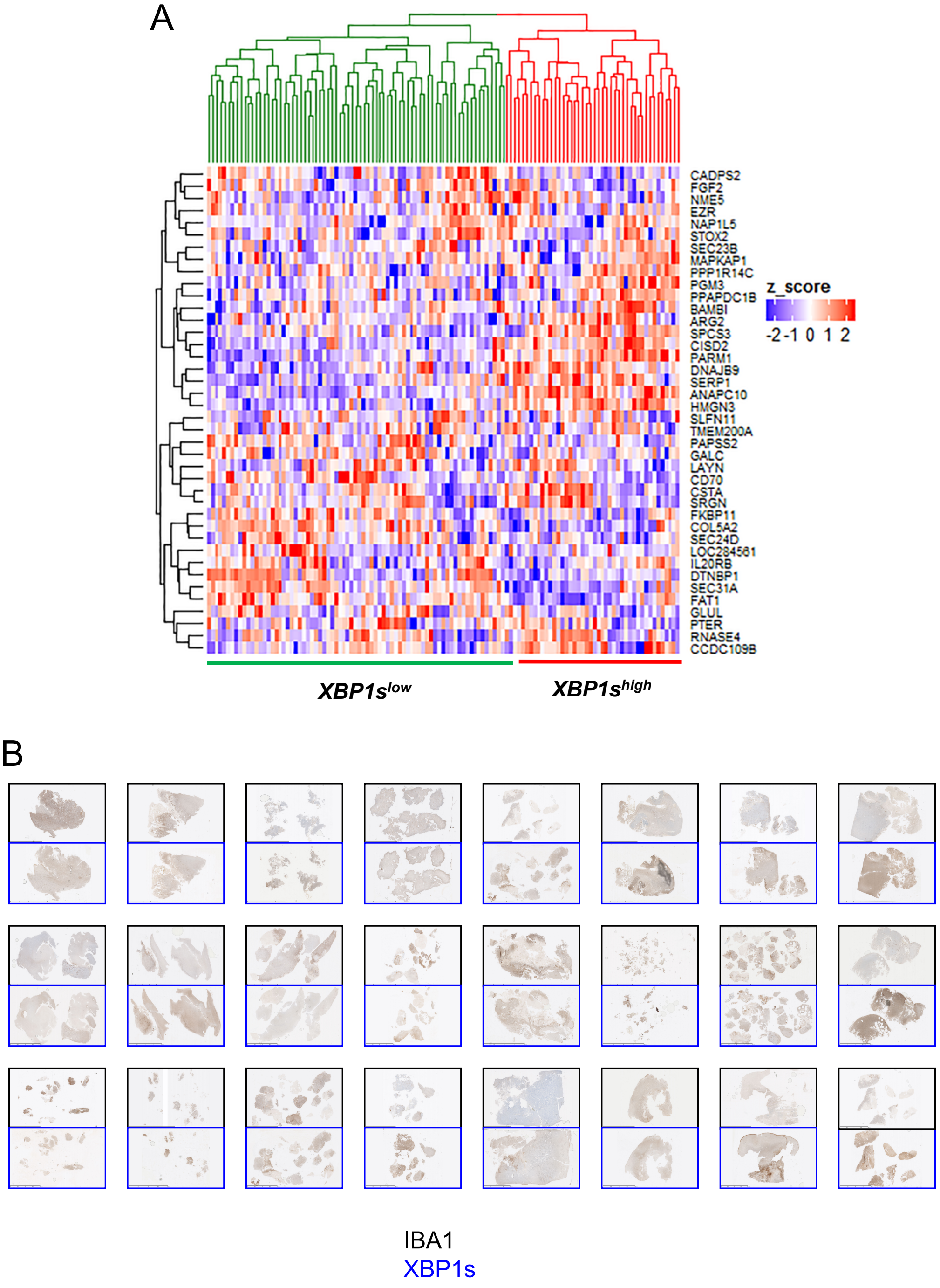
A
CADPS2
FGF2
NME5
EZR
NAP1L5
STOX2
SEC23B
MAPKAP1
PPP1R14C
PGM3
PPAPDC1B
BAMBI
ARG2
SPCS3
CISD2
PARM1
DNAJB9
SERP1
ANAPC10
HMGN3
SLFN11
TMEM200A
PAPSS2
GALC
LAYN
CD70
CSTA
SRGN
FKBP11
COL5A2
SEC24D
LOC284561
IL20RB
DTNBP1
SEC31A
FAT1
GLUL
PTER
RNASE4
CCDC109B
z_score
-2 -1 0 1 2
XBP1s^low
XBP1s^high
B
IBA1
XBP1s

Figure S6

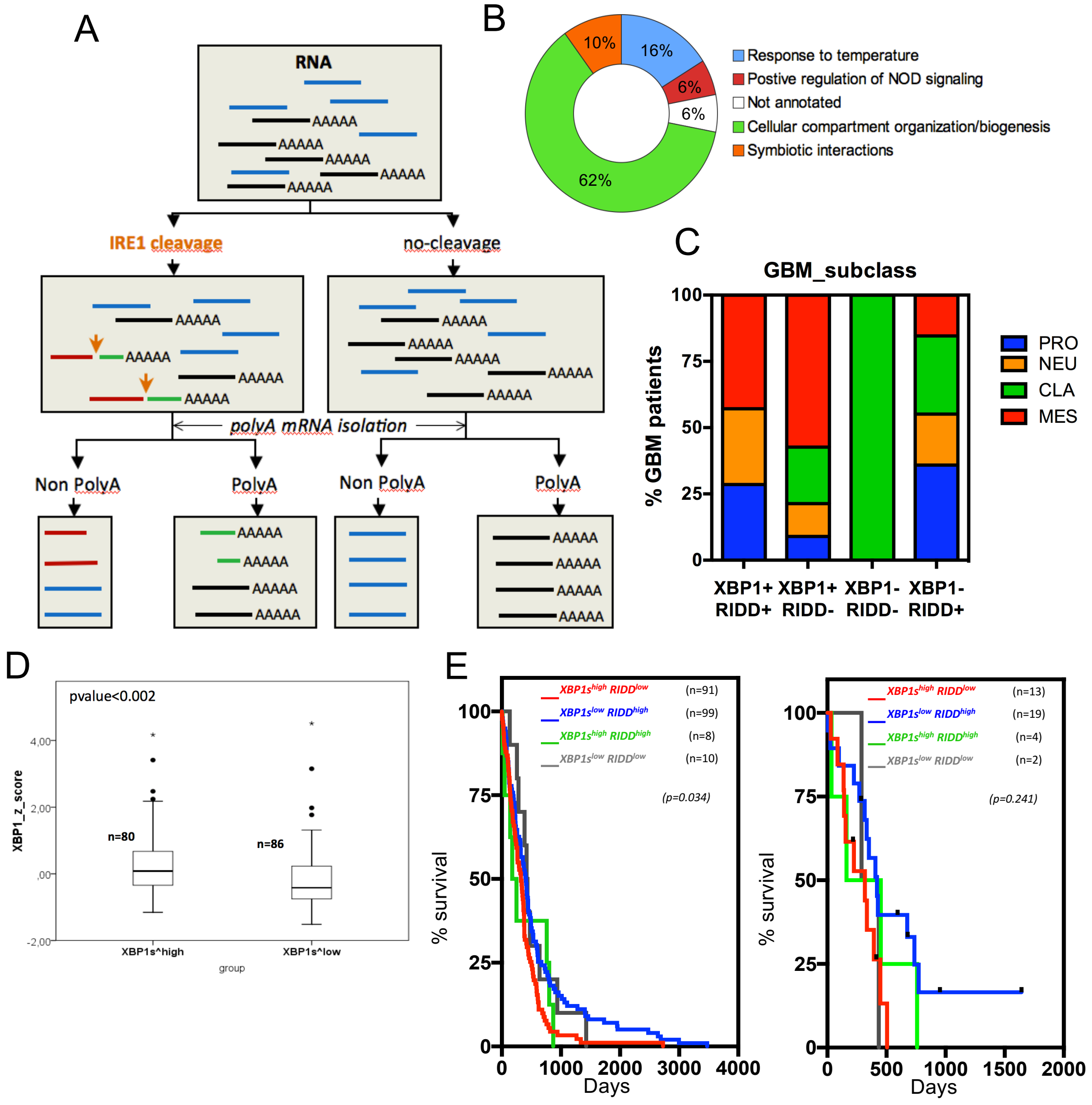
A
RNA
IRE1 cleavage
no-cleavage
polyA mRNA isolation
Non PolyA
PolyA
B
16%
6%
6%
62%
10%
Response to temperature
Postive regulation of NOD signaling
Not annotated
Cellular compartment organization/biogenesis
Symbiotic interactions
C
GBM_subclass
% GBM patients
PRO
NEU
CLA
MES
XBP1+ RIDD+
XBP1+ RIDD-
XBP1- RIDD-
XBP1- RIDD+
D
pvalue<0.002
XBP1_z_score
n=80
n=86
XBP1s^high
XBP1s^low
group
E
% survival
Days
(p=0.034)
(p=0.241)
(n=91)
(n=99)
(n=8)
(n=10)
(n=13)
(n=19)
(n=4)
(n=2)

Figure S7

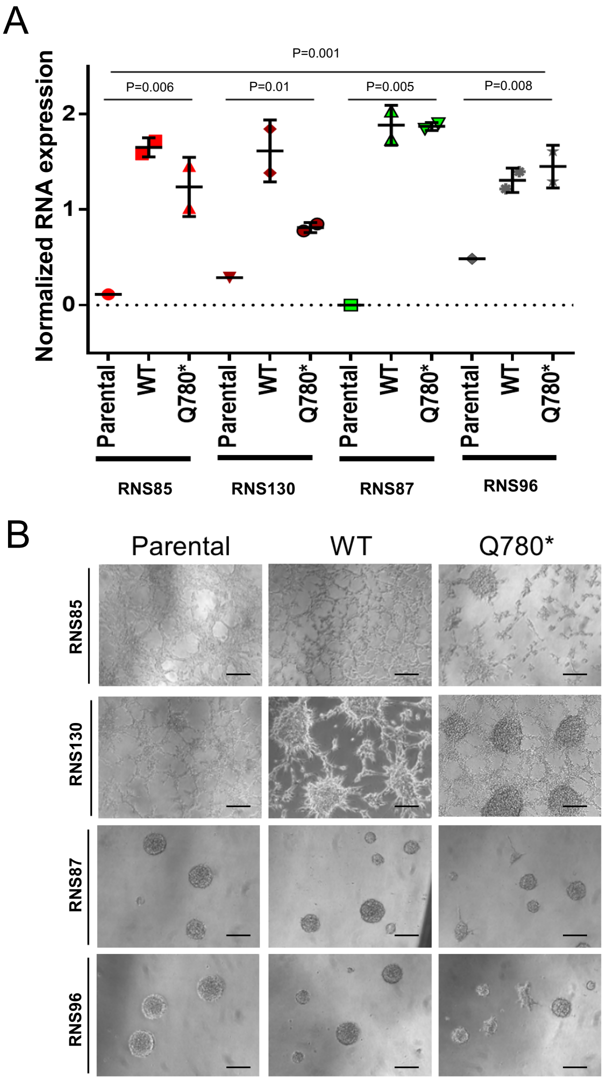
A
P=0.001
P=0.006
P=0.01
P=0.005
P=0.008
Normalized RNA expression
2
1
0
Parental
WT
Q780*
Parental
WT
Q780*
Parental
WT
Q780*
Parental
WT
Q780*
RNS85
RNS130
RNS87
RNS96
B
Parental
WT
Q780*
RNS85
RNS130
RNS87
RNS96

Figure S8
